# Case report: Combination therapy for hepatocellular carcinoma with inferior vena cava or right atrial tumor thrombus in the era of combined targeted and immunotherapeutic agents

**DOI:** 10.3389/fonc.2024.1470374

**Published:** 2024-12-11

**Authors:** Yubin Hai, Tingting Lin, Guangyi Wang, Xiaodong Sun, Lan Wang, Yuying Hai, Saisai Chen, Xiaoju Shi

**Affiliations:** ^1^ Department of Hepatobiliary and Pancreatic Surgery I, General Surgery Center, The First Hospital of Jilin University, Changchun, China; ^2^ Department of Hepatobiliary and Pancreatic Surgery II, General Surgery Center, The First Hospital of Jilin University, Changchun, China; ^3^ School of Stomatology, Jilin University, Changchun, China; ^4^ Department of Neurology, Stroke Center, The First Hospital of Jilin University, Changchun, China

**Keywords:** hepatocellular carcinoma, tumor thrombus, hepatectomy, targeted and immunotherapeutic drugs, case report

## Abstract

Primary liver cancer, predominantly hepatocellular carcinoma (HCC), is a leading cause of cancer-related mortality. Tumor thrombus (TT) in the inferior vena cava (IVC) or right atrium (RA) significantly worsens prognosis. We present four cases of male patients (average age 57) with HCC and TT extending into the IVC/RA, treated at our center. All underwent hepatectomy and TT resection, with targeted therapy (lenvatinib) and immunotherapy (sintilimab) administered post-operatively. Case 1 involved a 59-year-old male who had a right hepatectomy and TT resection in the IVC, followed by targeted therapy and immunotherapy, and is currently alive 74 months post-treatment. Case 2, a 48-year-old male, had a right hepatic lobectomy and TT resection in the IVC/RA, followed by liver transplantation 54 months postoperatively, with no recurrence. Case 3, a 66-year-old male, underwent a left hepatectomy and TT resection in the IVC, remaining disease-free 27 months postoperatively. Case 4, a 55-year-old male, received 15 cycles of combined targeted and immune therapy, followed by left hepatectomy and TT resection in the IVC/RA, with no recurrence 22 months postoperatively. Surgical resection combined with targeted and immunotherapy may enhance survival in advanced HCC patients with TT in the IVC/RA. Further studies are required to corroborate these findings.

## 1 Introduction

In the 21st century, the global incidence and mortality of cancer are rapidly increasing. According to the latest data from GLOBOCAN 2020 on primary liver cancer, there are approximately 905,677 new cases and 830,180 deaths annually worldwide. This makes primary liver cancer the sixth most common malignancy (4.7%) and the third-highest cause of cancer-related mortality (8.3%) (1). Primary liver cancer comprises hepatocellular carcinoma (HCC) in 75%-85% of cases, intrahepatic cholangiocarcinoma (ICC) in 10%-15% of cases, and other rare types (1). Therefore, HCC is the most prevalent form of liver cancer (2). Surgical curative resection is the standard treatment for achieving long-term survival in HCC patients, with a 5-year survival rate as high as 80% (3–5). However, the overall survival (OS) of patients with advanced HCC is notably poor, with a 3-year survival rate falling below 42.4% (6).

In certain situations, advanced HCC can lead to the formation of tumor thrombus (TT) in the portal vein and hepatic vein. Although TT rarely extends into the inferior vena cava (IVC) or right atrium (RA), when it occurs, the incidence rates are approximately 3.8% and 2.0%, respectively (7–9). TT extension into the IVC or RA may result in systemic metastasis and sudden death, attributed to pulmonary embolism or tricuspid valve embolism with TT. Reports indicate that the median survival times (MST) for untreated patients range from 2 to 5 months (9–14). Currently, there is no established treatment strategy for this type of patient. Advances in surgical techniques and perioperative management have made liver resection a relatively safe treatment option (15). However, the technical demands, associated high surgical risks, and a significantly elevated rate of postoperative complications greatly limit the implementation of this surgery.

With the gradual development of targeted therapies and the exploration of immune checkpoint inhibitors, particularly in the form of combination therapies, the treatment approach for HCC has entered a new stage (16, 17). For advanced HCC patients who are not suitable for surgery, systemic treatments like sorafenib and lenvatinib are recommended as first-line therapies (18). Innovative immunotherapies, encompassing immune checkpoint inhibitors targeting programmed cell death protein-1 (PD-1), cytotoxic T lymphocyte antigen 4 (CTLA-4), or its ligand programmed cell death-ligand 1 (PD-L1), have been integrated into the treatment regimens for advanced HCC (19). These advancements play a pivotal role in elevating survival rates and reshaping the overall therapeutic landscape for HCC. Importantly, they instill newfound optimism for patients with HCC and accompanying TT in the IVC/RA.

However, the effectiveness of surgical treatment in conjunction with targeted and immunologic drugs remains uncertain. In this context, we present four cases of HCC and TT in the IVC/RA who underwent surgical resection. These individuals underwent targeted therapy and immunotherapy after the surgical procedure. The primary objective of this study is to assess the impact of the combination of surgical resection with targeted and immune therapies on the prognosis for this specific tumor subtype.

## 2 Case presentation

In our center, a total of four patients underwent surgery under these circumstances, demonstrating the following characteristics: 1) diagnosis of HCC; 2) presence of IVC tumor thrombus; 3) no extrahepatic metastasis; 4) no other primary tumor lesions; 5) intact tumor without rupture; 6) child-Pugh classification and residual liver volume suitable for surgical resection. They underwent surgical resection of TT combined with hepatectomy. These patients were all male with an average age of 57 years. Among them, 2 patients presented with type III, 1 with type II, and 1 with type I. For a comprehensive overview of patient characteristics, please refer to **Table 1**.

**Table 1:** Detailed characteristics of the four patients.

Case	Case1	Case2	Case3	Case4
Age	59	48	66	55
Sex	Male	Male	Male	Male
Etiology	HBV	HBV	HCV	HBV
Quantification of virus	2.2×10^2^IU/L	2×10^6^IU/L	2.21×10^6^IU/L	<20IU/L
AFP before surgery	46.51ng/L	>800ng/L	20.58ng/L	348.7ng/L
Past treatment	sV and sVI resection, intervention	No	No	15 courses of Lenvatinib in combination with Sintilimab
Tumor location	Right lobe of the liver, IVC	Right lobe of the liver, IVC, RA	Left lobe of the liver, IVC	Left-right junction of the liver, IVC, RA
Child-Pugh (score, grade)	6, A	6, A	6, A	5, A
ICG-R15	7.1%	6.7%	12.2%	26.7%
BCLC stage	C	C	C	C
TT type	I	III	II	III
Surgical procedure	TT in IVC Right hepatectomy plus caudate lobectomy	TT in IVC and RA Right hepatectomy	TT in IVC Left hepatectomy	TT in IVC and RA Left hepatectomy
IVC reconstruction	Yes	Yes	Yes	Yes
Extracorporeal circulation	No	Yes	No	Yes
Pathological type	HCC	HCC	HCC	HCC
Pathological differentiation	Moderate	Moderate	Moderate-poor	Moderate
Tumor volume	11cm*11cm*8cm	5.5cm*2.8cm*2.5cm	16cm*13cm*11.5cm	7.5cm*3cm*3cm
Surgical margin	–	–	–	–
MVI	M1	M1	M1	M0
AJCC- TNM	T4N0M0	T4N0M0	T4N0M0	T4N0M0
Postoperative targeted therapy	Lenvatinib	Lenvatinib	Lenvatinib	Lenvatinib
Postoperative Immunotherapy	Sintilimab	Sintilimab	Sintilimab	Sintilimab
PFS, months	45	38	27	22
Recurrence	Yes	Yes	No	No
Postoperative survival, months	74	69	27	22

TT, tumor thrombus; IVC, inferior vena cava; RA, right atrium; HCV, hepatitis C virus; HBV, hepatitis B virus; AFP, alpha-fetoprotein; PFS, progression free survival; HCC, hepatocellular carcinoma; BCLC, Barcelona Clinic Liver Cancer; MVI, Microvascular invasion; TNM, Tumor-Node-Metastasis; AJCC, the American Joint Committee on Cancer.

### 2.1 Case 1

**Figure 1:**
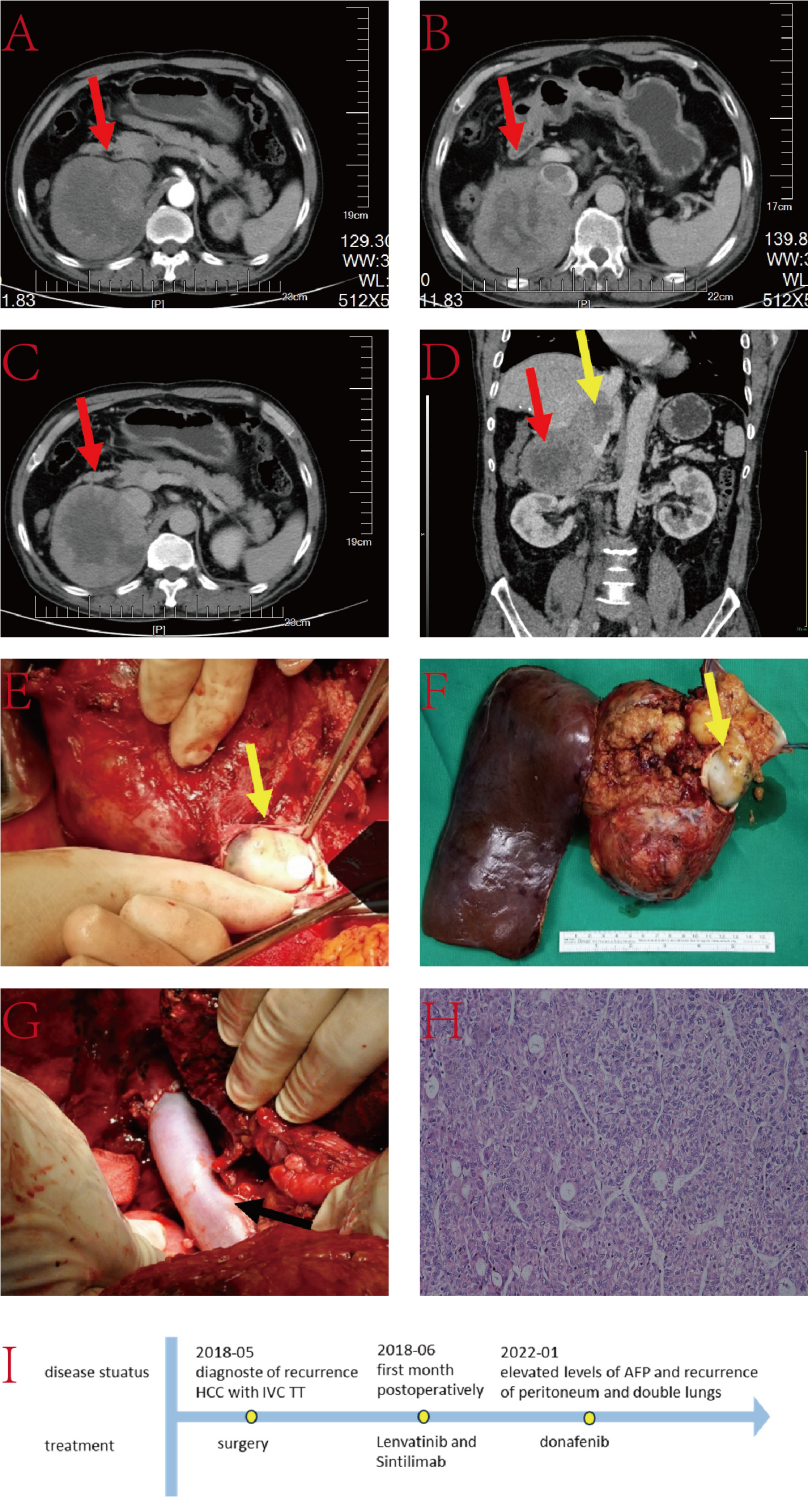
**(A–C)** Liver computed tomography (CT) images displaying the tumor lesion in the liver (red arrow); **(D)** Coronal plane reconstruction by CT illustrating the position of the tumor lesion (red arrow) and TT in the IVC (yellow arrow); **(E)** Intraoperative view revealing the TT in the IVC (yellow arrow); **(F)** Pathological specimen exhibiting the tumor lesion and TT (yellow arrow); **(G)** Reconstructed IVC with Donation after circulatory death (DCD) vessels (black arrow); **(H)** Histopathologic examination of the hepatic mass and liver tissue (hematoxylin and eosin staining, ×20); **(I)** The timeline scheme of the major clinical events of the patient since diagnosis.

The patient is a 59-year-old male with a history of smoking, alcohol consumption, and hepatitis B infection, without regular antiviral treatment. His viral load was measured at 2.2×10^2^IU/L. Alpha-fetoprotein (AFP) was 46.51ng/L, and he has no family history of genetic disorders. He underwent SV and SVI resection in our hospital 26 months ago, followed by an interventional treatment 24 months ago. Subsequent postoperative follow-up revealed the presence of a TT in the IVC and an occupancy in the right lobe of the liver (**Figure 1**). After a comprehensive evaluation, he underwent right hepatectomy, caudate lobe resection, removal of IVC TT, and IVC reconstruction.

Intraoperative ultrasound revealed that the IVC tumor thrombus was located between the hepatic vein and renal vein levels. After marking the ischemic line, right hepatic lobe and caudate lobe resection were performed under blockage of the first hepatic hilum and lower IVC. The IVC was blocked at both the proximal hepatic vein and distal renal vein levels. The IVC was then incised, and the TT along with the invaded portion of the IVC was completely excised. Subsequently, the resected IVC was reconstructed. The entire procedure lasted nearly 10 hours without any major surgery-related complications. He was discharged 18 days after surgery.

### 2.2 Case 2

**Figure 2:**
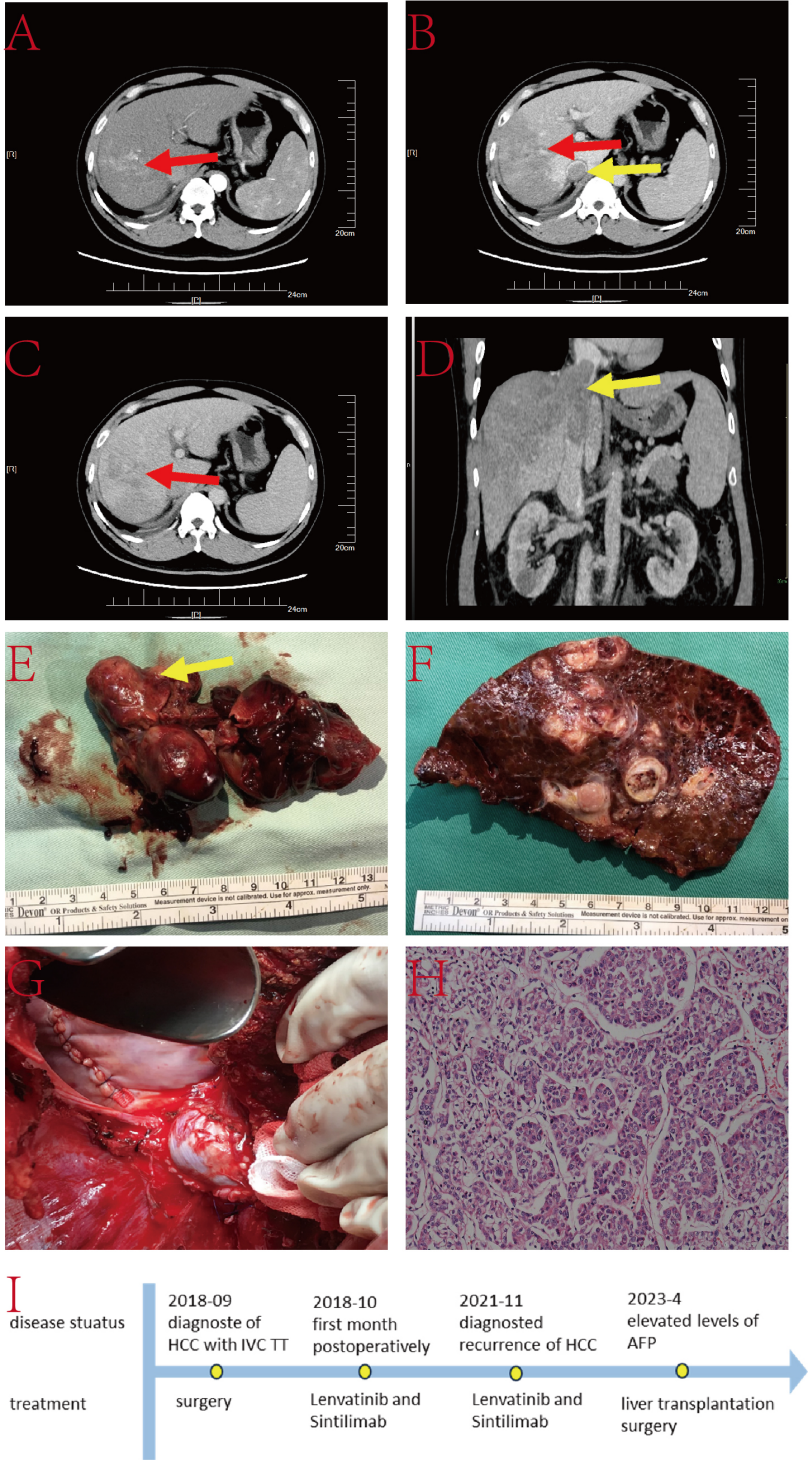
**(A–C)** Liver CT images illustrating the tumor lesion in the liver (red arrow) and TT in the IVC (yellow arrow); **(D)** Coronal plane reconstruction by CT indicating the position of the TT in the IVC (yellow arrow); **(E)** TT in the IVC and RA (yellow arrow) with a partial view of the IVC; **(F)** Pathological specimen displaying the tumor lesion; **(G)** Reconstructed IVC using DCD vessels and reconstructed autologous pericardium; **(H)** Histopathologic examination of the hepatic mass and liver tissue (hematoxylin and eosin staining, ×20); **(I)** The timeline scheme of the major clinical events of the patient since diagnosis.

The patient is a 48-year-old male with a 3-month history of anorexia and weight loss. He has a history of hepatitis B infection, without regular antiviral treatment. His viral load is 2×10^6^IU/L. AFP was exceeding 800ng/L, and he has no family history of genetic disorders. Abdominal CT revealed a substantial lesion in the right lobe of the liver, with the TT extending to the IVC, RA, and the right branch of the portal vein (**Figure 2**). Treatment for this case involved right hepatic lobectomy combined with the surgical removal of the TT in the IVC and RA under extracorporeal circulation.

Following the marking of the ischemic line during surgery, the liver tissue was dissected along the ischemic line under blockage of the first hepatic hilum and lower IVC. The chest was then opened, and the mediastinum and diaphragm around the IVC were incised to expose the heart. An extracorporeal circulation pathway was established. The RA was opened, and the right hepatic vein was transected at its root to remove the right hepatic lobe. A vertical 8 cm incision was made on the surface of the IVC, and the IVC TT and the invaded IVC were completely excised. The RA incision was then closed, and the resected IVC was reconstructed. The entire procedure lasted nearly 9 hours without any major surgery-related complications. The patient was discharged 14 days postoperatively. Follow-up MRI at 38 months postoperatively showed signs of malignancy in the SIV nodules. Subsequent evaluations confirmed that the patient met the Milan criteria for liver transplantation. Therefore, the patient underwent liver transplantation surgery at the 54rd postoperative month.

### 2.3 Case 3

**Figure 3:**
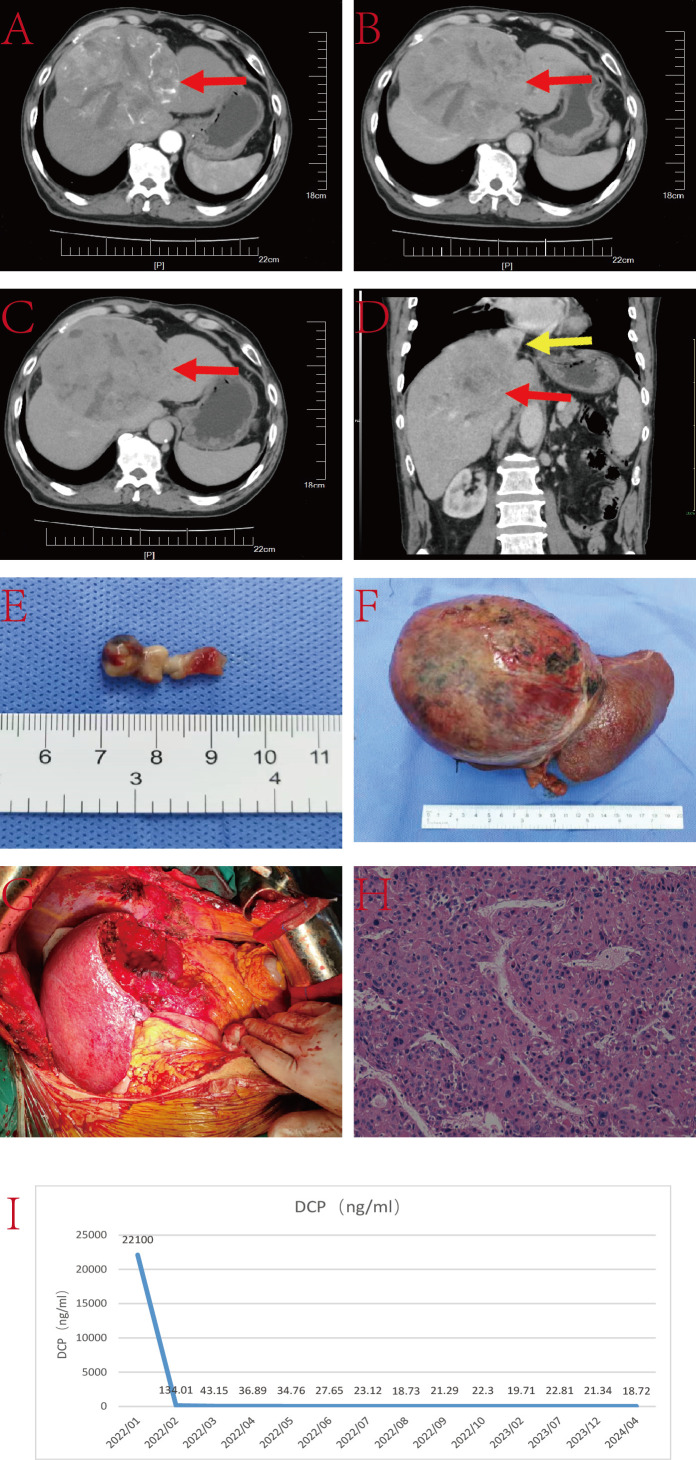
**(A–C)** Liver CT images displaying the tumor lesion in the liver (red arrow) and TT in the IVC (yellow arrow); **(D)** Coronal plane reconstruction by CT illustrating the tumor lesion (red arrow) and the position of the TT in the IVC (yellow arrow); **(E)** TT; **(F)** Pathological specimen showcasing the tumor lesion; **(G)** Operative field post-resection of the tumor lesion and TT; **(H)** Histopathologic examination of the hepatic mass and liver tissue (hematoxylin and eosin staining, ×20); **(I)** DCP level.

The patient is a 66-year-old male with a history of hepatitis C infection, without regular antiviral treatment. His viral load is 2.21×10^6^IU/L. AFP was 20.58ng/L, and he has no family history of genetic disorders. He was diagnosed with TT in the IVC and a lesion in the left lobe of the liver (**Figure 3**). The treatment for this case included a left hepatectomy combined with surgical resection of the TT in the IVC.

Under intermittent blockage of the first hepatic hilum, the left hepatic lobe was dissected along the ischemic line. After opening the chest, the mediastinum and diaphragm around the IVC were incised, and the pericardium was opened to fully expose the heart. Intraoperative ultrasound carefully located the IVC TT. Under blockage of the IVC, a longitudinal incision was made on the anterior wall of the IVC corresponding to the TT, fully exposing the thrombus. The left hepatic lobe and TT were excised together. Subsequently, IVC reconstruction and pericardial repair were performed. The entire surgery lasted nearly 6 hours without any major surgery-related complications. The patient was discharged 17 days postoperatively.

### 2.4 Case 4

**Figure 4:**
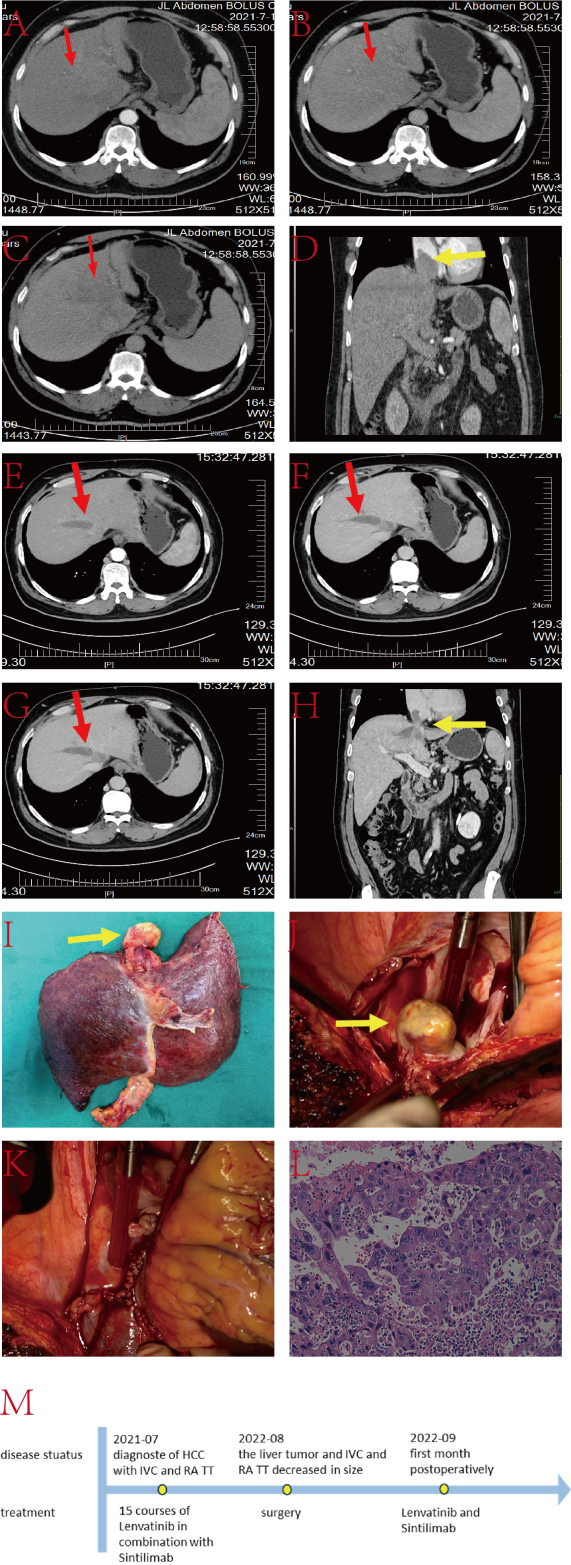
**(A–C)** Liver CT images displaying the tumor lesion in the liver (red arrow); **(D)** Coronal plane reconstruction by CT illustrating the position of the TT in the IVC and RA (yellow arrow); **(E–G)** Sequential images depicting the reduction in tumor volume after 15 courses of targeted and immunologic drugs.; **(H)** Coronal plane reconstruction by CT illustrating the position of the TT in the IVC and RA (yellow arrow) after 15 courses of targeted and immunologic drugs, with a reduced size.; **(I)** Pathological specimen showcasing the TT (yellow arrow), tumor lesion and part of IVC; **(J)** TT in IVC and RA (yellow arrow); **(K)** Reconstructed IVC using DCD vessels and reconstructed autologous pericardium; **(L)** Histopathologic examination of the hepatic mass and liver tissue (hematoxylin and eosin staining, ×20); **(M)** The timeline scheme of the major clinical events of the patient since diagnosis.

The patient is a 55-year-old male with a history of hepatitis B infection, on regular antiviral treatment, with a viral load of less than 20IU/L. AFP was 348.7ng/L. He has no family history of genetic disorders. His condition presented as a lesion at the junction of the left and right liver lobes, with TT involving the IVC, RA and the left branch of the portal vein (**Figure 4**). Due to limitations in the remaining liver volume and metabolic demands, immediate tumor resection was considered impractical. After receiving 15 cycles of combined targeted and immune therapy (lenvatinib combined with sintilimab) in the oncology department, imaging showed a reduction in tumor size from 7.1 cm × 4.0 cm to 6.5 cm × 2.7 cm, and a decrease in the length of the tumor thrombus from 7 cm to 4 cm.

Upon multidisciplinary team consensus, it was determined that left hepatectomy combined with surgical resection of the TT in the IVC and RA under extracorporeal circulation would be the optimal course of action for the patient. During the operation, the second hepatic hilum, retrohepatic IVC, and first hepatic hilum were fully exposed and dissected. Intraoperative ultrasound showed a TT in the left branch of the portal vein, with no thrombus in the right branch or main trunk. Thrombectomy was performed along with resection of the ventral segment of the right anterior lobe and the left hepatic lobe. The liver was split until the retrohepatic IVC was fully exposed. TT was identified in the middle hepatic vein and IVC at the second hepatic hilum, extending above the diaphragm and into the atrium. After opening the chest and pericardium to fully expose the heart, cardiopulmonary bypass was established. With the first hepatic hilum and infrahepatic IVC clamped, an incision was made in the right atrium to remove the IVC tumor thrombus, the resected liver tissue, and the intracardiac mass together. The right atrium and IVC were then repaired. The entire surgical procedure lasted approximately 11 hours without any major surgery-related complications. The patient was discharged 25 days postoperatively.

The tumors and TT were both completely resected. All four patients underwent IVC reconstruction, with reconstruction vessels from DCD donors in Cases 1, 2, and 4. These patients began regular antiviral therapy immediately after surgery and received a combination treatment of Levotinib (oral 12 mg, once daily) and Sintilimab. Fortunately, during follow-up, their AFP levels and imaging results showed no signs of recurrence, and they did not exhibit any clinical signs or symptoms of relapse. Their relapse-free survival times were 45 months, 38 months, 27 months, and 22 months, respectively. The OS times were 74 months, 69 months, 27 months, and 22 months, respectively. The median recurrence-free survival (RFS) time and MST were 32.5 months and 48 months, respectively.

## 3 Discussion

HCC is a highly malignant tumor known for its aggressive nature. It commonly metastasizes to regional lymph nodes, lungs, or bones and exhibits invasion into vasculature (20). As previously mentioned, the occurrence of HCC with TT in the IVC or RA is approximately 3.8% and 2.0%, respectively. Globally, the incidence of IVC and RA involvement ranges from 1.4% to 4.9% (21). Anatomically, TT is clinically categorized into three types based on its location relative to the heart: type I, where the TT is located below the diaphragm in the IVC (referred to as the inferior hepatic type); type II, where the TT is located above the diaphragm in the IVC, but still outside the RA (known as the superior hepatic type); and type III, where the TT is located above the diaphragm and has entered the RA (referred to as the intracardiac type) (15).

According to the Barcelona Clinic Liver Cancer (BCLC) staging system, HCC with TT in the IVC/RA is classified as stage C, representing an extremely poor prognosis in the advanced stage (7, 8). The recommended treatment approach by the European Association for the Study of the Liver (EASL) clinical practice guidelines is “systemic therapy” (18). Studies have reported the MST of 17.8 months for surgical patients, compared to 10.1 months for non-surgical patients (22). Radical resection has demonstrated MSTs of up to 19.0 months, with higher 5-year survival rates than non-surgical approaches (13).

The observed benefits in surgical outcomes may be attributed to the gradual advancements in surgical techniques. At our center, we conduct these surgeries based on the ongoing progress in liver transplantation and extracorporeal circulation technology. However, it is essential to emphasize the associated risks linked with such surgical operations, as patients may face potential complications, such as pulmonary embolism or perioperative bleeding. Drawing from our experience, we employ a combined thoracoabdominal incision, and the extracorporeal circulation device is meticulously prepared in advance. The procedure involves complete resection of the hepatic tumor and embolus causing IVC blockage. Circulation is subsequently re-established using either autologous vessels or DCD vessels.

Li et al. have outlined a surgical guide for the treatment of HCC with TT in the IVC/RA (15). To ensure surgical safety, they emphasized performing surgery exclusively on patients with Child-Pugh A classification and good hepatic reserve for HCC (8). For type I, in cases involving complete hepatic blood flow blockage, a longitudinal incision is made in the IVC, enabling the complete removal of both the primary intrahepatic lesion and TT. In type II, an incision can be made in the diaphragm anterior to the IVC TT through a median sternotomy and thoracotomy, exposing the TT above the diaphragm. Subsequently, the tumor and TT are removed, and the IVC wall, pericardium, and diaphragm are sutured. Type III cases necessitate a combination of cardiothoracic and hepatobiliary surgery under extracorporeal circulation. Blood flow is bypassed to the ascending aorta after ex vivo oxygenation. The RA is incised, and the entire TT is excised.

Two of our cases underwent combined thoracoabdominal surgery under extracorporeal circulation, involving the complete resection of TT and liver masses. However, due to severe invasion of the IVC, all four cases underwent IVC reconstruction, with three cases utilizing DCD vessels for reconstruction. It is noteworthy that, in the literature we reviewed, there were no reports on the use of DCD vessels for IVC reconstruction. This approach contrasts with the use of artificial vessels, which are costly and anticoagulant-dependent. The use of DCD vessels is both cost-effective and of high quality, making it a promising option for patients undergoing this procedure at qualified transplant centers.

The guidelines from the European Association for the Study of the Liver (EASL), the American Association for the Study of Liver Diseases (AASLD), and the Japan Society of Hepatology (JSH) all emphasize that, for advanced HCC patients with well-preserved liver function (Child-Pugh class A), first-line therapeutic options commonly include lenvatinib, sorafenib, atezolizumab plus bevacizumab combination therapy, and camrelizumab plus apatinib (23–25). Additionally, Chinese guidelines also advocate the use of sintilimab in combination with IBI305 (a bevacizumab biosimilar) as a first-line treatment for unresectable or metastatic HCC (26).

Historical literature reports that, for unresectable HCC patients, the MST with sorafenib ranges from 10.3 to 13.6 months, with a median progression-free survival (PFS) of 3.6 to 4.3 months (27–29). Lenvatinib, on the other hand, demonstrates a MST between 12.3 to 12.8 months, with a median PFS of 6 to 7 months (28, 30). A study indicated that patients receiving lenvatinib had improved OS, PFS, objective response rate, and disease control rate (DCR) compared to those treated with sorafenib (31). A randomized phase III study also demonstrated superior efficacy of lenvatinib over sorafenib (OS 15.2 months vs. 10.5 months, PFS 7.0 months vs. 4.5 months). Patients receiving lenvatinib showed a 36% reduction in the risk of death, a 29% reduction in the risk of progression, higher response rates and higher disease control rates (32).

In recent years, there has been rapid development in systemic therapy for advanced HCC, with promising outcomes achieved by novel systemic treatments. Literature reports indicate that monotherapy with pembrolizumab in previously untreated advanced HCC results in a median PFS of 4 months and the MST of 17 months (33). In the analysis of the IMbrave150 study, the MST in the atezolizumab plus bevacizumab group was superior to sorafenib (19.2 months vs. 13.4 months, and the median PFS (6.9 months vs. 4.3 months) (34). Additionally, in the analysis of the ORIENT-32 study, the combination of sintilimab and IBI305 (a bevacizumab biosimilar) significantly improved the MST (not reached vs. 10.4 months) and extended the median PFS (4.6 months vs. 2.8 months) (35). A phase 1b study of pembrolizumab in combination with lenvatinib also yielded positive results: with an overall response rate of 36.0%, disease progression rate of 7.0%, PFS of 8.6 months, and OS of 22.0 months (36). Furthermore, the latest data from the phase 3 LEAP-002 trial (NCT03713593) suggests that the combination of lenvatinib and pembrolizumab provides a survival advantage for patients with advanced HCC (median PFS 8.2 months vs. 8.0 months; MST 21.1 months vs. 19.0 months) (37). These studies collectively underscore the importance of immunotherapy in the management of advanced liver cancer patients.

Moreover, postoperative adjuvant therapy is particularly crucial for patients with a high risk of recurrence in liver cancer. A study demonstrated that postoperative immune checkpoint inhibitors (ICIs) combined with targeted therapy showed significantly higher RFS in univariate analysis. This suggests that postoperative adjuvant ICI combined with targeted therapy may reduce the recurrence of HCC in patients with most risk factors (38). Results from a phase III randomized controlled trial (IMbrave 050) indicated that atezolizumab combined with bevacizumab significantly improved postoperative RFS in patients (39). Another study suggested that postoperative anti-PD-1 antibody treatment in high-risk recurrent HCC patients demonstrated better postoperative 1-year, 2-year, 3-year, and 4-year OS rates (93.1% vs. 85.3%, 86.8% vs. 70.2%, 78.2% vs. 47.7%, 51.1% vs. 30.0%) and postoperative 1-year, 2-year, 3-year RFS rates (81.7% vs. 68.4%, 77.0% vs. 47.7%, 52.3% vs. 25.8%) compared to the non-anti-PD-1 antibody group (40). Additionally, postoperative adjuvant therapy (PAT) with tyrosine kinase inhibitors (TKI) and anti-PD-1 antibodies can improve surgical outcomes for HCC patients at high risk of recurrence. The PAT group showed 1-year and 2-year RFS rates of 82.1% and 40.0%, respectively, and 54.2% and 25.1%. The corresponding 1-year and 2-year OS rates were 95.4% and 69.8% and 84.3% and 55.5% (40). Liver resection surgery combined with postoperative lenvatinib treatment can provide additional survival benefits for patients (28). Taking into consideration the guidance from comprehensive guidelines and the reported patient benefits in previous research literature, we have administered postoperative treatment to patients, including targeted therapy, immunotherapy, or a combination of targeted and immunotherapeutic agents.

The therapeutic outcomes of various treatment strategies for HCC patients with TT in IVC/RA were reviewed in the literature. The median time to recurrence (TTR) after radical resection is 3 to 9.8 months, and the MST ranges from 16.7 to 30.8 months (9, 13, 14). Patients undergoing radiotherapy have an MST of 17.4 months, while TACE results in an MST of 10.9 months (41, 42). Systemic or hepatic arterial infusion chemotherapy achieves an MST of 15.4 months, whereas chemotherapy combined with radiotherapy results in an MST of 7.9 months (22, 42). Best supportive care yields an MST of 5.9 months (22).Combination therapies, including TACE with chemotherapy, TACE plus external beam radiotherapy (EBRT), and TACE with sorafenib, show superior efficacy compared with TACE alone (43–45). Survival rates for patients undergoing liver resection are higher than those receiving TACE, and the MST for surgical resection is superior to that of the EBRT group (15.3 months vs 11.7 months) (46, 47). Patients treated with TACE combined with lenvatinib and sintilimab achieve a median MST of 17.3 months and a median PFS of 13.0 months (48). Radiotherapy combined with targeted therapy provides an MST of 15.8 months and a median PFS of 4.2 months (49).

Overall, aggressive treatments—including surgery, radiotherapy, interventional therapy, systemic therapy, and multimodal approaches—demonstrate improved survival outcomes (21). Among these, surgical resection offers the greatest survival benefit (50).The median RFS and MST of the cases at our center were 32.5 months and 48 months, respectively. Compared to the survival benefit reported in the literature, our case showed a significant survival benefit.

With the advancement of targeted and immunotherapy research, the role of the tumor microenvironment in tumor progression has gained increasing attention. The tumor microenvironment (TME) is composed of immune cells, fibroblasts, cytokines, and extracellular matrix, which dynamically interact with tumor cells to create an immune-suppressive environment (51). This environment promotes the expression of angiogenic factors such as VEGF and immunosuppressive cytokines like TGF-β, accelerating vascular infiltration while weakening antitumor immune responses (52). These processes play a critical role in the initiation and progression of HCC. Recent advances in targeted therapies, including the combination of TKIs and ICIs, have shown significant progress in modulating the TME. For instance, the combination of lenvatinib and pembrolizumab normalizes blood vessels and reawakens exhausted T cells, thereby enhancing antitumor efficacy through TME modulation (51). It is noteworthy that Case 4 showed significant shrinkage of TT, and postoperative pathology indicated severe cancer cell necrosis within the lesion. This further confirms the effectiveness of the combination of targeted and immunological drugs for unresectable advanced HCC and the modulation of TME.

For late-stage HCC patients with TT in the IVC/RA who are not suitable for surgery, a combination of targeted and immunological drugs may successfully convert such cases into operable patients. The amalgamation of aggressive surgical resection and combination of targeted and immunological drugs may confer benefits to HCC patients with TT in IVC/RA. Our clinical cases emphasize the importance of involving relevant experts in a multidisciplinary team and ensuring timely treatment intervention to develop the optimal treatment plan for such patients.

In general, the survival benefits obtained in the four cases at our center exceeded those reported in previous literature. Cases 3 and 4 had a relatively short postoperative period, emphasizing the importance of continuous close follow-up for ongoing assessment. It is essential to emphasize that our center has treated only four patients in this manner, with only one patient receiving preoperative conversion therapy with a combination of targeted and immune therapies. Therefore, further relevant case studies are necessary to strengthen our understanding of the treatment approaches for patients in such conditions.

## Data Availability

The original contributions presented in the study are included in the article/supplementary material. Further inquiries can be directed to the corresponding author.
